# Intravital Two-photon Imaging of Ca^2+^ signaling in Secretory Organs of Yellow Cameleon Transgenic Mice

**DOI:** 10.1038/s41598-018-34347-1

**Published:** 2018-10-26

**Authors:** Kai Jin, Toshihiro Imada, Shigeru Nakamura, Yusuke Izuta, Erina Oonishi, Michiko Shibuya, Hisayo Sakaguchi, Takahiro Adachi, Kazuo Tsubota

**Affiliations:** 1 Department of Ophthalmology, Keio University School of Medicine, 35 Shinanomachi, Shinjuku-ku, Tokyo 160-8582 Japan; 2 Department of Immunology, Medical Research Institute, Tokyo Medical and Dental University, 1-5-45 Yushima, Bunkyo-ku, Tokyo 113-8510 Japan

## Abstract

Intracellular calcium ([Ca^2+^]i) signaling regulates physiological functions in most cells. In secretory organs, such as the pancreas, salivary gland, and lacrimal gland (LG), [Ca^2+^]i elevation in acinar cells triggers fluid secretion, which plays vital roles in the maintenance of functional health across the life-course. It is important to understand the secretory mechanism of secretory organs, but lack of analytic systems available for living animals limits the scope of research to gain deeper insights into the precise mechanism of secretion. We established an intravital imaging system for specific cell types of secretory organs to monitor the [Ca^2+^]i changes using mouse line expressing Yellow Cameleon 3.60, a genetically encoded Ca^2+^ indicator. Elevation of [Ca^2+^]i in specific cell types of secretory organs could be monitored after cholinergic stimulation *ex vivo* and intravitally. We found that a marked attenuation of LG [Ca^2+^]i response to cholinergic stimulation was induced under pathological conditions by postganglionic denervation. Intravital Ca^2+^ imaging in secretory organs will broaden our understanding of the cellular mechanisms in animal models of secretory diseases.

## Introduction

Intracellular Ca^2+^ signaling plays important roles in regulating a wide variety of cellular physiological processes^1–3^. Intracellular Ca^2+^ concentration ([Ca^2+^]i) is regulated by intracellular release from the endoplasmic reticulum store or influx through a variety of Ca^2+^ channels in response to stimulation by neurotransmitters and a variety of hormones^1,2^.

In secretory organs, such as the pancreas, salivary gland (SG), and lacrimal gland (LG), [Ca^2+^]i elevation in the secretory acinar cells is the key trigger for secretion of a mixture of water and proteins synthesized in the acinar cells^3–5^. The fluid mixture produced by secretory organs is secreted onto the epithelial surface out of the organs, and is necessary for maintenance of epithelial homeostasis and body metabolism^3–5^. The dysfunction of secretory organs leads to various diseases, such as diabetes, dry mouth, and dry eye^5–8^, by poorly understood mechanisms. Evaluation of intracellular Ca^2+^ signaling under normal physiological and pathological conditions is an effective approach to elucidate the mechanisms underlying the diseases caused by dysfunction of secretory organs.

Synthetic Ca^2+^ indicators, such as Fura-2, Fluo4 and Indo 1, have been extensively used for monitoring intracellular Ca^2+^ signaling^9–11^. Although these synthetic Ca^2+^ indicators exhibit high sensitivity to Ca^2+^ and rapid response kinetics, the disadvantages include limitation of loading to specific cell types within an intact tissue and insufficient intracellular retention^10^.

In recent studies of Ca^2+^ signaling, genetically encoded Ca^2+^ indicators (GECIs) were preferred for measuring Ca^2+^ signaling in precisely targeted intracellular locations, cell-specific intravital analysis, and extended time-lapse monitoring without fluorescence leakage^10^. Yellow Cameleon (YC), a widely used GECI, employs fluorescence resonance energy transfer (FRET) from cyan fluorescent protein (CFP) to yellow fluorescent protein (YFP) in response to [Ca^2+^]i elevation^12^. Upon binding of free Ca^2+^, the Ca^2+^ responsive element calmodulin (CaM) of YC alters the efficiency of the two fluorescent proteins CFP and YFP. The other well-recognized GECI is GCaMPs, a single fluorophore probe based on a circularly permuted green fluorescent protein, their monitoring of Ca^2+^ signaling is depending on fluorescence intensity. Compared to GCaMPs, YCs have less dynamics range and photostability but the advantages of them are less sensitive to motion artifact and expression level differences because of rationing of two fluorescent proteins^13,14^.

Previously, a transgenic mouse line conditionally expressing YC3.60 was established to monitor the long-term, spatiotemporal Ca^2+^ signaling in living animals^13,15^. This mouse line has been demonstrated to be useful in monitoring the Ca^2+^ signaling in lymphoid tissues (spleen, Peyer’s patches, and bone marrow) and intestinal gut epithelial cells^13,15^. However, no studies have demonstrated intravital imaging of Ca^2+^ signaling in specific secretory organs of YC3.60 transgenic mice.

In this manuscript, we describe a useful and efficient visualization system to monitor the Ca^2+^ signaling within secretory organs in a cell-type-specific manner using YC3.60 transgenic mice in combination with two-photon microscopy. This visualization system opens a wide range of new possibilities in the study of intravital secretory activities or behaviors.

## Results

### Localization of the YC3.60 probe in secretory organs isolated from YC3.60 transgenic mice

**Figure 1:**
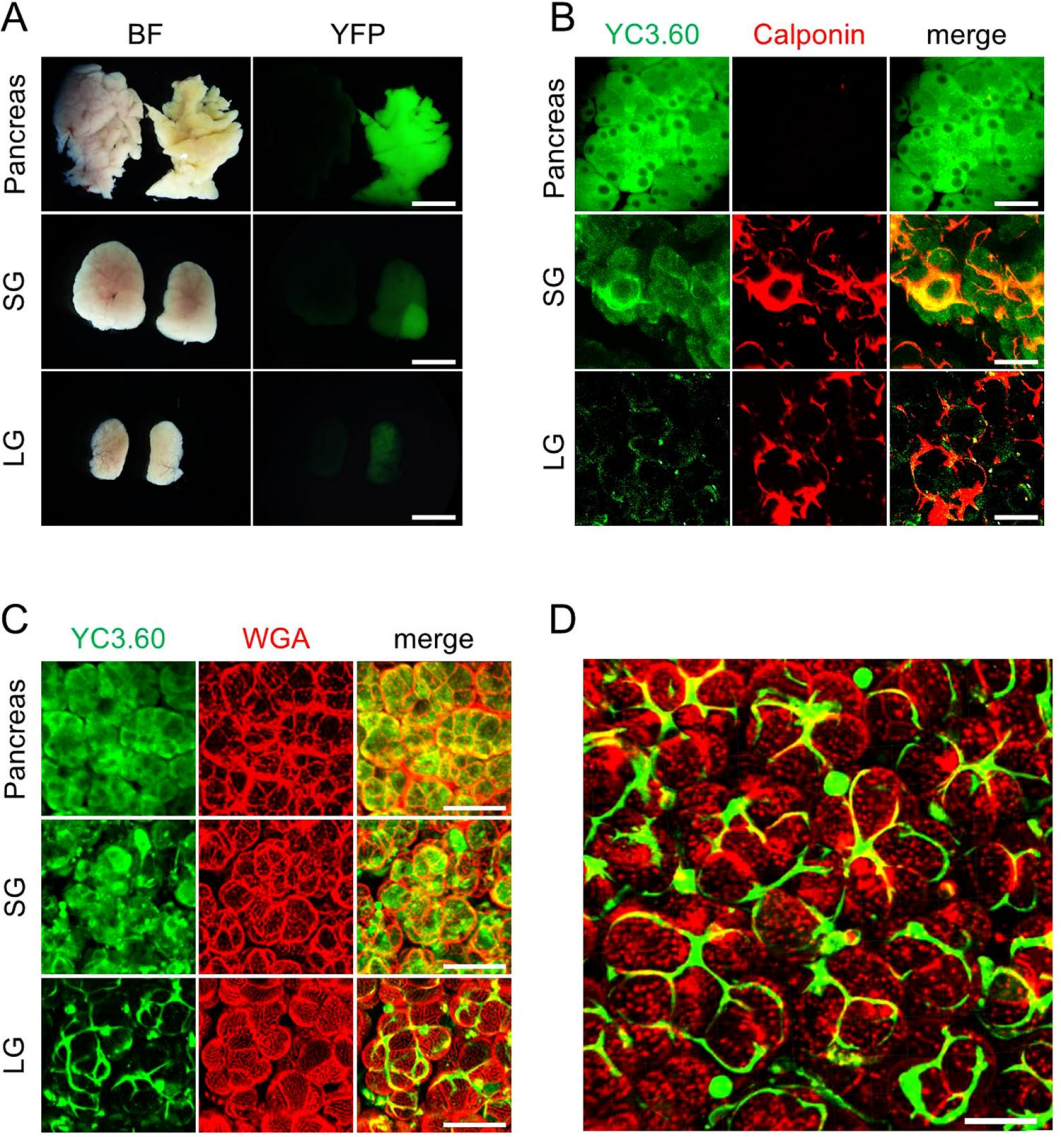
Expression of the YC3.60 probe in secretory organs of YC3.60 transgenic mice. (**A**) Bright-field (BF) and fluorescence (YFP) images of isolated secretory organs from wild type mouse (left) and YC3.60 transgenic mouse (right). (**B**,**C**) Localization of YC3.60 probe in secretory organs. Secretory organs from YC3.60 transgenic mice were immunostained with antibodies for calponin, a myoepithelial cell marker (**B**), and was stained with wheat germ agglutinin (WGA), a marker for cellular surface (**C**). Each panel shows the pancreas (upper), salivary gland (SG, middle), and lacrimal gland (LG, lower) (**A**–**C**). (**D**) Three-dimensional (3D) imaging of YC 3.60 localization in the lacrimal gland. Green and red indicates the YC3.60 probe and cellular surface, respectively. Scale bar is 4 mm (**A**), 200 µm (**B**,**C**), and 20 µm (**D**).

To determine the suitability of YC3.60 for Ca^2+^ imaging in secretory organs, we examined the tissue morphology and distribution of YC3.60 expression in various secretory organs. There were no obvious differences in tissue morphology between YC3.60 and wild-type mice. (Fig. 1A left column).

Ubiquitous green fluorescence was observed in all secretory organs we examined in YC 3.60 mice. Wild-type mice showed no fluorescence (Fig. 1A right column).

Acinar cells are polarized epithelial cells that play an essential role in the production of secretory components^3,4^. Myoepithelial cells (MEC) surrounding the basal surface of acinar cells are stellate-shaped cells and have a contractile function, which helps expelling secretory components from acinar cells to the excretory duct^16–18^.

Secretory organs are composed primarily of acinar cells, and MEC have also been found in almost all secretory organs with notable exception of the pancreas^19^.

To examine the localization of the YC3.60 probe in secretory organs, we performed immunofluorescent staining of calponin (Fig. 1B) and wheat germ agglutinin (WGA) staining (Fig. 1C) of secretory organs isolated from YC3.60 transgenic mice.

In the pancreas, calponin-positive cells were not observed and fluorescence from the YC3.60 probe was observed in the cytoplasm of acinar cells. In the salivary gland, calponin-positive cells which are stellate in shape were observed. The fluorescence of the YC3.60 probe was localized in calponin-positive cells and parts of the cytoplasm of acinar cells. Calponin-positive cells were also observed in the lacrimal gland (LG). The fluorescence of YC3.60 probes co-localized with calponin-positive cells in the LG.

Figure 1D and Supplementary movie 1 showed the localization of YC3.60 probes in the LG. It clearly portrayed the discontinuous network of stellate YC3.60-positive cells covering the basal surface of the acinar cells.

Our results of the distribution of MEC positively stained with the anti-calponin antibody in various secretory organs corresponded with previous reports^16–18^. These results indicate that localization of the YC3.60 probe uniquely exhibits a cell-type-specific pattern in secretory organs of YC3.60 transgenic mice.

### Evaluation of FRET ratio dynamics in pancreases isolated from YC3.60 transgenic mice

The autonomic nervous system regulates the secretory functions of the pancreas, SG, and LG^17,20^. Parasympathetic stimulation increases outflow of pancreatic fluid, saliva, and tears through the intracellular Ca^2+^ mobilization due to the activation of muscarinic acetylcholine receptors (mAChRs) in the pancreas, SG, and LG, respectively^3,21,22^.

**Figure 2:**
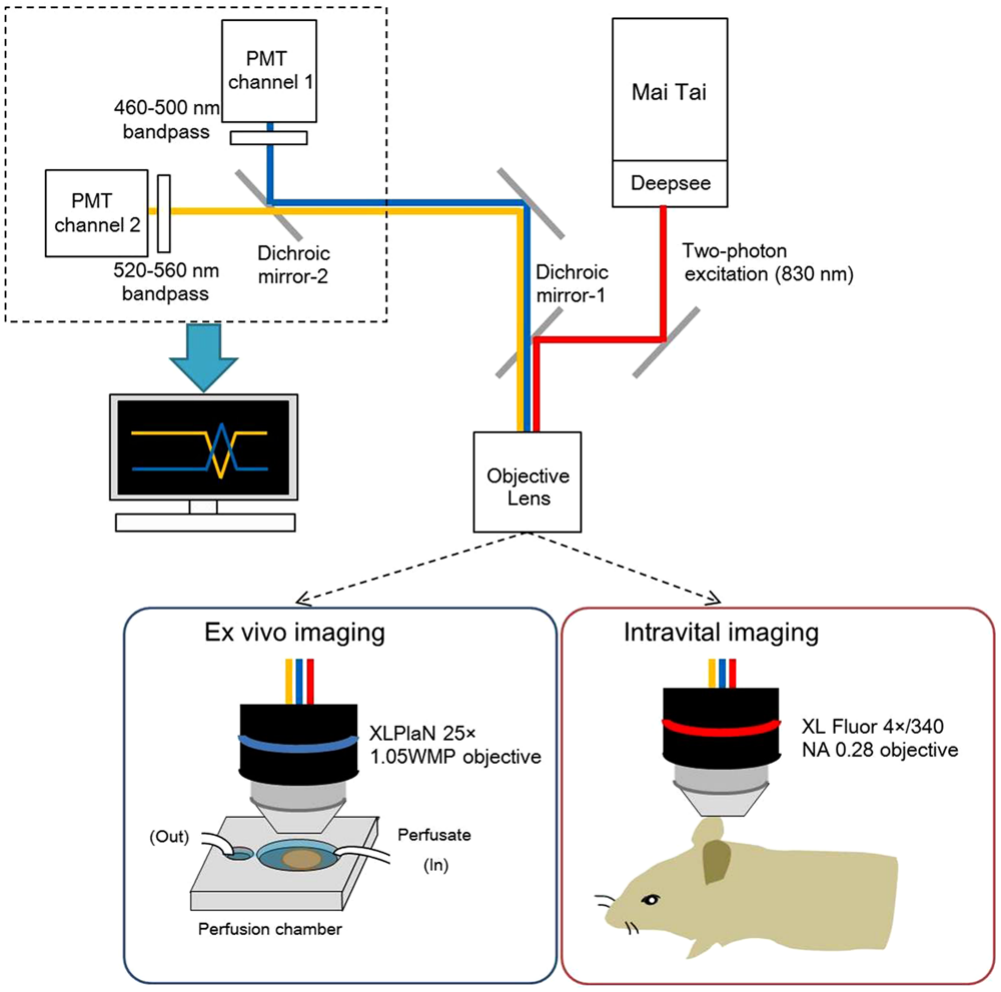
Schematic representation of our *ex vivo* and intravital Ca^2+^ imaging system. (**A**) femtosecond laser beam from a MaiTai HP Ti:Sapphire laser is coupled with an Olympus FV1200 MPE system with a BX61WI body. A MaiTai® DeepSeeTM unit is placed in the front of MaiTai to compensate for velocity dispersion. Two-photon excitation was performed at a wavelength of 830 nm. The objective lens (XL PlaN 25× and XL Fluor 4×/340, was used to obtain a wide range of images at high resolution for *ex vivo* imaging and intravital imaging, respectively. The two-photon excited fluorescence images of CFP and YFP were obtained in separate channels through dichroic mirrors and emission filters, BP460-500 and BP520-560, respectively. Emission was simultaneously detected through a band-pass filter for rhodamine (>575 nm) and GFP (510–550 nm).

To confirm whether the genetically encoded YC3.60 probe can be used in spatiotemporal Ca^2+^ imaging in secretory organs *ex vivo* and intravitally, we established the *ex vivo* and intravital Ca^2+^ imaging systems. Figure 2 shows a schematic representation of *ex vivo* and intravital two-photon Ca^2+^ imaging system.

We initially evaluated the changes in FRET ratios induced by the calcium ionophore ionomycin; cyclopiazonic acid (CPA) which is an inhibitor of the endoplasmic reticulum Ca^2+^ ATPase pump^23^ and the mAChR agonist acetylcholine (ACh) using isolated pancreases from YC3.60 transgenic mice by using our *ex vivo* imaging system.

**Figure 3:**
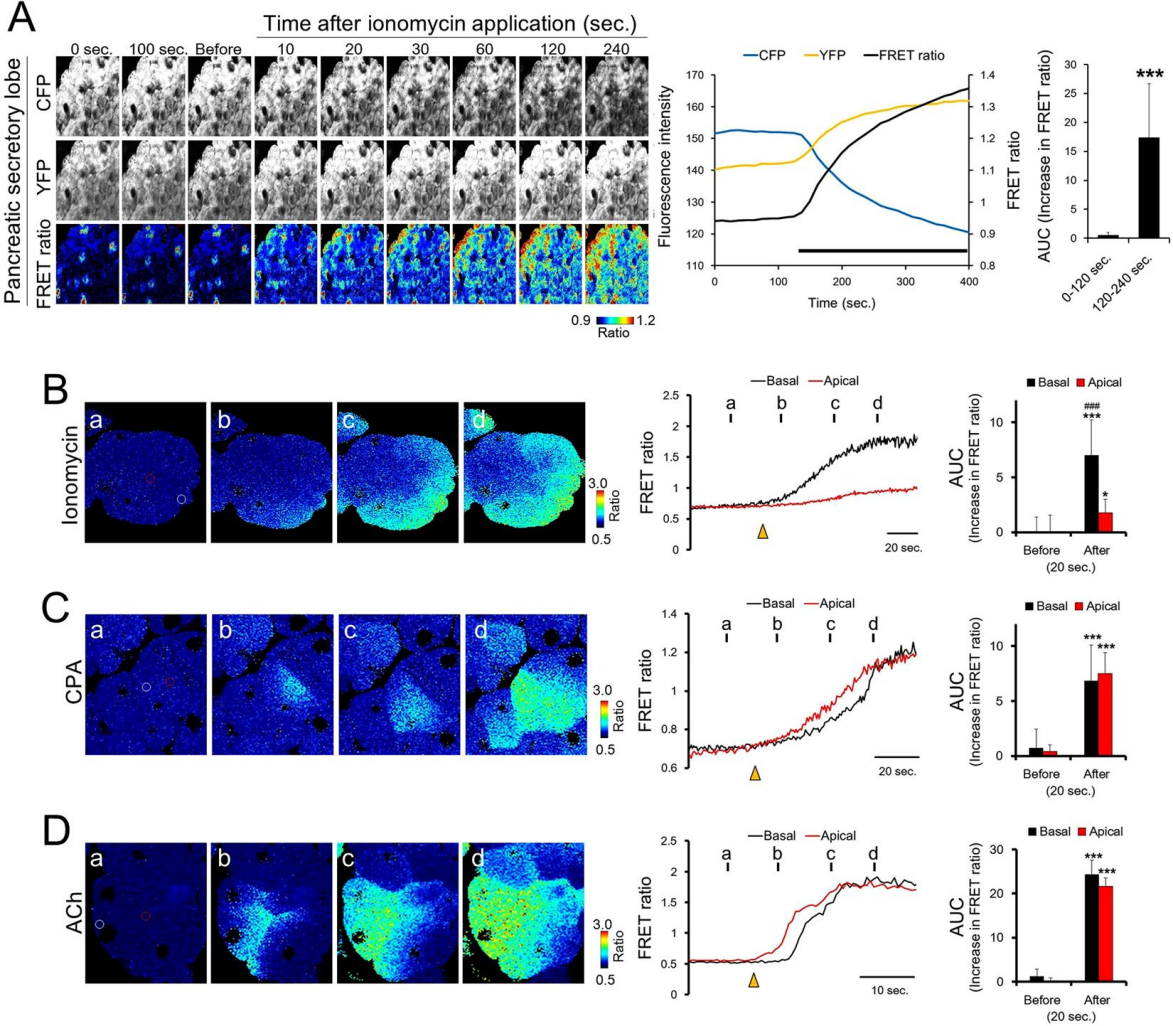
FRET ratio-based imaging of pancreases isolated from YC3.60 transgenic mice. (**A**) Change in the FRET ratio in the secretory lobes of the pancreas by ionomycin stimulation. Each panel shows two-photon excited fluorescence images of CFP (upper panel), YFP (middle panel), and pseudo-colored images of the FRET ratio (lower panel) before and after stimulation with ionomycin. The line chart indicates the changes in fluorescence intensity of CFP, YFP, and the FRET ratio. Bold black bars under the line chart indicate the presence of ionomycin. The bar chart indicates the AUC of the FRET ratio. (**B**–**D**) Comparative analysis of the differences in FRET ratio changes by (**B**) ionomycin, (**C**) CPA, and (**D**) ACh between the basal and apical regions of pancreatic acinar cells. Left panels show the pseudo-color image of the change in the FRET ratio. White and red circles indicate the basal and apical regions, respectively. The line chart shows the changes in the FRET ratio in the basal (black) and apical regions (red) of pancreatic acinar cells. The yellow arrowheads indicate the time at which each drug was applied to the pancreas. Each bar chart indicates the AUC of the FRET ratio 20 s before and after stimulation. Values are the mean ± SD of 4−5 independent experiments. ***P < 0.001 for 0−120 s (**A**) and before (**B**–**D**). ^###^P < 0.001 versus apical (**B**).

Figure 3A (the upper panels) showed the image dynamics of CFP and YFP, and pseudo color images of ratiometric FRET ratio in secretory lobes of pancreas.

There were no changes in the fluorescence of CFP and YFP before ionomycin stimulation (0 and 100 sec, baseline). 10 seconds after the ionomycin stimulation, a gradual decrease in the extent of CFP fluorescence and an increase in YFP fluorescence were observed when compared to that before stimulation. The fluorescence changes of CFP and YFP were sustained long after the application of ionomycin to the isolated pancreas. Ratiometric FRET images remained unchanged before ionomycin stimulation and began to change in response to ionomycin stimulation. Corresponding to the dynamics of CFP, YFP, and FRET images, ionomycin stimulation induced a notable increase in the FRET ratio accompanying inverse changes in CFP and YFP intensities (Fig. 3A line chart). The value of the AUC (area under the curve, in the line chart) was significantly higher after ionomycin stimulation (120−240 sec) compared with that before stimulation (0−120 sec).

Previous reports demonstrated that an Ach-induced Ca^2+^ wave propagates from apical to basal regions in pancreatic acinar cells loaded with Fura2, a synthetic Ca^2+^ indicator^24^. The Ca^2+^ wave occurred rapidly in the apical region at a speed of approximately 10 µm/second, and propagated towards the basal region^24^. Therefore, we analyzed the dynamics of the FRET ratio in pancreatic acinar cells during ionomycin, CPA, and ACh treatment under high-speed acquisition to investigate whether the intracellular Ca^2+^ wave induced by each drug was detected in the YC3.60 transgenic mice.

After ionomycin stimulation, FRET ratio elevation occurred in the basal region of pancreatic acinar cells and gradually propagated towards the apical regions (Fig. 3B). The elevation of the FRET ratio induced by ionomycin was significantly lower in the apical region than that in the basal region of pancreatic acinar cells. Ionomycin is known to cause selective influx of Ca^2+^ from the extracellular medium^25,26^. To maintain the Ca^2+^ concentration in the cytoplasm, excessive Ca^2+^ is stored in intracellular Ca^2+^ stores, such as the endoplasmic reticulum and mitochondria, or effluxed to the extracellular space^2,3^. Thus, our results of a higher elevation of FRET ratio in the basal than apical regions of acinar cells can be explained on the basis of ionomycin-activated Ca^2+^ influx pathway and the efflux activity of excessively elevated intracellular Ca^2+^.

CPA triggered the onset of FRET ratio elevation at the apical region and the elevation was propagated across the cytoplasm towards the basal region (Fig. 3C). There was no difference in the elevation of FRET ratio induced by CPA between the basal and the apical regions. CPA is known to cause the intracellular Ca^2+^-release from endoplasmic reticulum by inhibiting Ca^2+^ uptake^23,27^. Dissimilarity of FRET ratio dynamics in pancreatic acinar cells induced by ionomycin and CPA is thought to be due to the difference in mechanism underlying intracellular Ca^2+^ mobilization.

After ACh stimulation, the onset of FRET ratio elevation occurred at the apical region and propagated in an apical-to-basal direction (Fig. 3D). This result is consistent with a previous report^24^. Supplementary movie 2–4, and 5 shows the changes in FRET ratio induced by ionomycin in the pancreatic secretory lobe, pancreatic acinar cells, SG, and LG, respectively.

These results support the availability of the genetically encoded YC3.60 probe as a useful tool for spatiotemporal Ca^2+^ imaging in pancreas.

### Evaluation of FRET ratio dynamics in the salivary and lacrimal glands isolated from YC 3.60 transgenic mice

**Figure 4:**
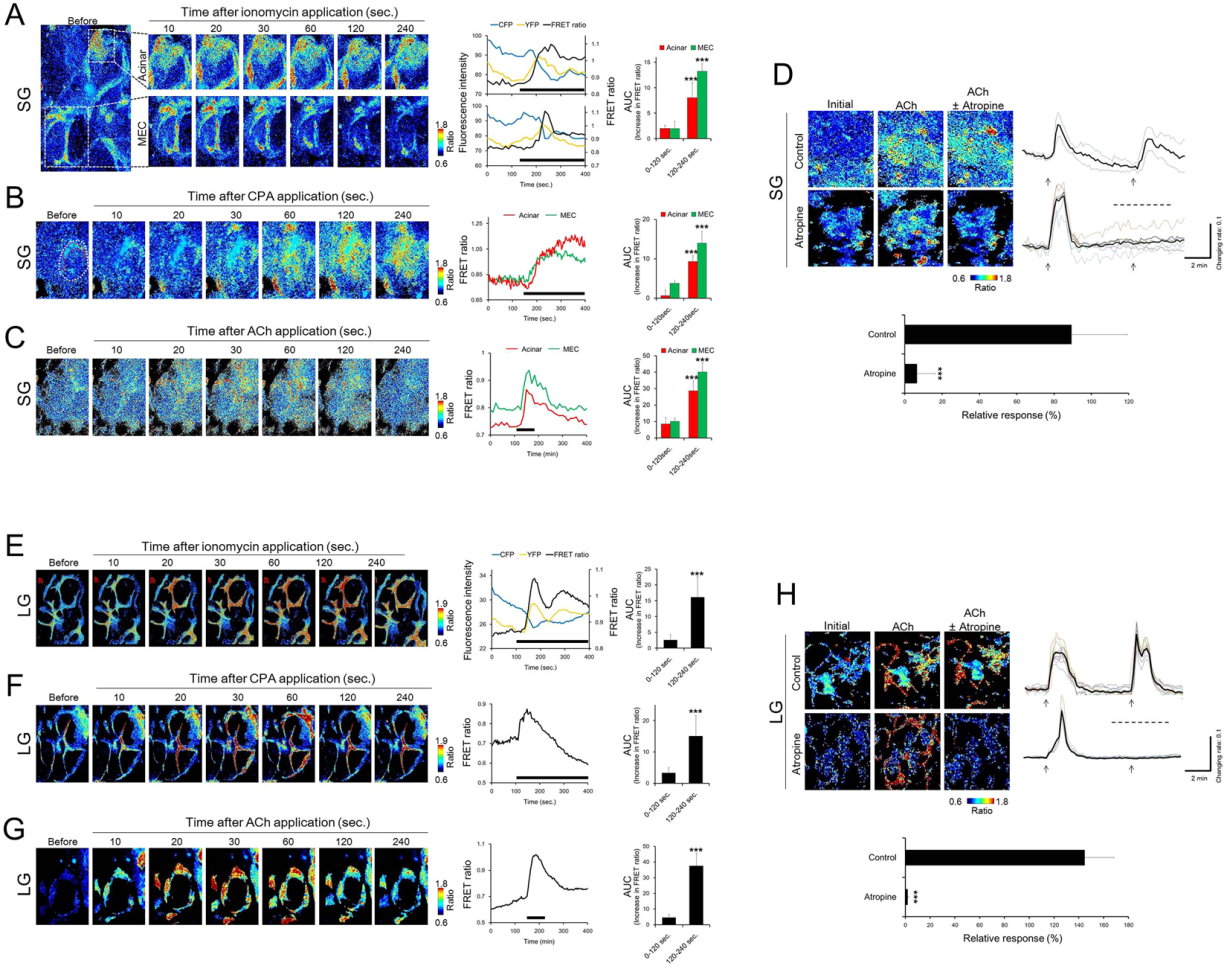
Changes in the FRET ratio dynamics in the salivary and lacrimal glands. (**A**–**C**) FRET ratio changes in the salivary gland induced by (**A**) ionomycin, (**B**) CPA, and (**C**) ACh. (**D**) Inhibitory effects of atropine on ACh-induced FRET ratio elevation in the salivary gland. (**E**–**G**) FRET ratio changes in the lacrimal gland induced by (**E**) ionomycin, (**F**) CPA, and (**G**) ACh. (**G**). Inhibitory effects of atropine on ACh-induced FRET ratio elevation in the lacrimal gland. Left (**A**–**C**, **E**–**G**) and upper panels (**D**,**H**) indicate the pseudo-color images of FRET ratio elevation. Line charts show the changes in fluorescence intensity of CFP, YFP, and the FRET ratio in acinar cells and myoepithelial cells in (**A**) the salivary and (**E**) lacrimal gland during ionomycin stimulation. Bar graphs show the AUC of the FRET ratio before (0−120 s) and after (120−240 s) stimulation with each drug. The middle line charts show the changes in the FRET ratio induced by CPA (**B**,**F**) and ACh (**C**,**G**) in the salivary gland and lacrimal gland. The black and bold bars under the line chart indicate the presence of each drug. The line charts on the right indicate the representative FRET ratio changes induced by ACh with (control) and without atropine. The arrow indicates the time at which ACh was applied to the secretory organs. The dotted lines over the line charts indicate the presence of atropine. Relative responses (bar chart) were calculated as a percentage of the decrease in the ACh-induced increase in the FRET ratio with the application of atropine relative to the stimulation induced by ACh alone. All data represent the mean ± SD, n = 4−5 independent experiments. ***P < 0.001 versus 0−120 s (**A**–**C**, **E**–**G**) or versus control (**D**, **H**).

Next, we analyzed the FRET ratio dynamics of acinar cells and their surrounding MEC in the SG. In acinar cells, the FRET ratio increased immediately after ionomycin stimulation and maintained elevated levels throughout ionomycin stimulation (Fig. 4A). In the MEC, the FRET ratio transiently increased and thereafter maintained a plateaued increase during ionomycin stimulation, indicating that the FRET ratio response induced by ionomycin in the MEC was different from that in acinar cells.

Intracellular Ca^2+^ in the acinar cells regulates the synthesis of secretory proteins and the exocytotic secretion of intracellular proteins^28,29^. In the myosin containing MEC, intracellular Ca^2+^ is necessary for contraction to generate direct physical force to stimulate the acinar cells. Therefore, differences between the FRET ratio responses of the two cell types are suspected to reflect the distinct functional properties of each cell type.

The onset of the initial increase in acinar cells and MEC occurred almost at the same speed. The AUC values of both acinar cells and MEC were significantly higher after ionomycin stimulation.

After the stimulation with CPA, the FRET ratios increased and plateaued in both MEC and acinar cells (Fig. 4B). The increase in the FRET ratio was significantly higher than that before CPA stimulation in both acinar cells and MEC.

Stimulation with ACh transiently increased the FRET ratio to a higher level (peak) in both acinar cells and MEC of SG, and the increased FRET ratio gradually returned to the baseline after removal of ACh (Fig. 4C). In both acinar cells and MEC, a significantly higher AUC value for the increase in FRET ratio was observed after ACh stimulation than before stimulation. This result was similar to the dynamics of the FRET ratio in acinar cells and MEC stimulated with ionomycin and CPA.

In presence of the mAChR antagonist atropine, the FRET ratio elevation induced by ACh was inhibited compared to the FRET ratio following ACh stimulation alone at the same dosage in the SG (Fig. 4D). The relative response significantly decreased due to the presence of atropine compared to the response in the absence of atropine (control).

We also analyzed the FRET ratio dynamics in the LG MEC. After stimulation with ionomycin, the FRET ratio in LG MEC transiently increased to a higher level, then dipped and returned to a sustained plateau. The AUC value of the LG MEC was significantly higher after ionomycin stimulation compared to before stimulation (Fig. 4E).

After the stimulation with CPA, the FRET ratio in the MEC of LG peaked and gradually returned to the baseline (Fig. 4F).

ACh stimulation induced a transient increase of the FRET ratio in the LG MEC, and the increased FRET ratio gradually returned to the baseline after removal of ACh (Fig. 4G). For the LG MEC, the FRET ratio dynamics observed after ACh stimulation corresponded with a previous report demonstrating that cholinergic stimulation induced a transient increase in [Ca^2+^]i in the MEC of Fura2-loaded LG from guinea pigs^17^. Additionally, atropine mostly inhibited FRET ratio elevation induced by ACh in the LG MEC compared to the control.

These data confirmed that YC3.60 enables the monitoring of Ca^2+^ responses to physiological stimulation in secretory organs.

### Intravital imaging analysis of intracellular Ca^2+^ signaling in secretory organs

Intravital imaging of transgenic mice carrying genetically encoded Ca^2+^ indicators (GECIs) using two-photon microscopy is an advantageous strategy to study the physiological function of specific organs when compared to the strategies using synthetic Ca^2+^ indicators, because GECIs allow for long-term and cell-type-specific imaging without potential damage to the target organs^12,13,15^.

To examine whether YC3.60 transgenic mice in combination with two-photon microscopy are useful in visualizing the intracellular Ca^2+^ signaling along with secretory function, we analyzed the relationship between Ca^2+^ mobilization in specific types of cells of secretory organs and fluid secretion from corresponding secretory organs following cholinergic stimulation intravitally. Bethanechol (Bet) is a parasympathomimetic choline carbamate that selectively stimulates muscarinic receptors without any effect on nicotinic receptors^30^. Unlike acetylcholine, Bet is not hydrolyzed by cholinesterase^31^. Therefore, it has a long duration of action in our intravital imaging analysis.

**Figure 5:**
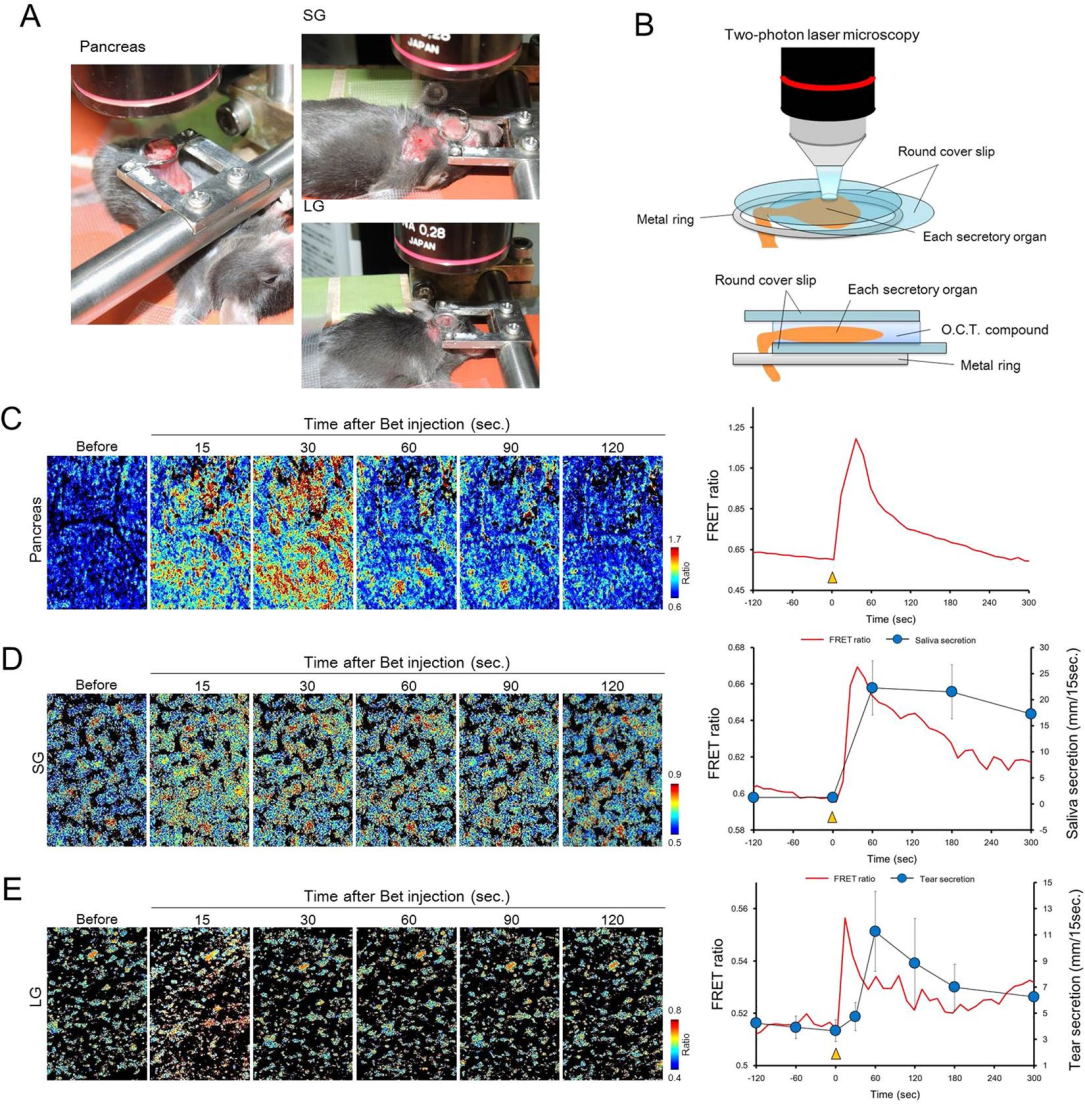
Intravital Ca^2+^ imaging of secretory organs in YC3.60 transgenic mice. (**A**) Photographs of intravital imaging of the pancreas (left), SG (right upper), and LG (right lower) of YC 3.60 transgenic mouse. (**B**) Schematic representation of the condition that each secretory organ is observed with two-photon microscopy. Lower schema indicates the side view. (**C**–**E**) Changes in the FRET ratio induced by intravenous injection of bethanechol (Bet) in the pancreas (**C**), SG (**D**), and LG (**E**). The left panels show the pseudo-color images of the FRET ratio. The line chart shows changes in the FRET ratio. Blue closed circles show the changes in saliva secretion (**D**) and tear secretion (**E**). Yellow arrowheads indicate the time at which Bet was intravenously injected into mice. Data represents the mean ± SD, n = 4−5 mice.

Figure 5A,B show the photographs and schematic representation of experimental set-up for intravital imaging of each secretory organ, respectively.

In the pancreas, the ratiometric FRET ratio image was unchanged before Bet stimulation and changed to a warm color 15 seconds after intravenous injection of Bet (Fig. 5C). The change in the FRET ratio gradually returned to the pre-stimulus level 120 seconds after Bet injection.

In the SG, Bet changed the FRET ratio image to a warm color 15 seconds after the injection (Fig. 5D). The FRET ratio image gradually recovered but maintained a relatively warm color 120 seconds after Bet injection.

In the LG, a change of the FRET ratio image was observed immediately after the injection of Bet (Fig. 5E). The warm colored FRET ratio image recovered 120 seconds after Bet injection.

Supplementary movie 6, 7, and 8 shows the change of the FRET ratio image in the pancreas, SG, and LG induced by intravenous injection of Bet.

Corresponding to the dynamics of ratiometric FRET images, intravenous injection of Bet induced dramatic increases in FRET ratio of the pancreas, SG, and LG (Fig. 5C–E, line chart). These results observed by intravital Ca^2+^ imaging of these secretory organs were similar to the results of *ex vivo* Ca^2+^ imaging.

Estimation of [Ca^2+^]i from observed FRET ratio was performed based on Hill equation according to previous report ^9,14^, and the intravital [Ca^2+^]i in pancreas, SG and LG was varied by Bet stimulation in the range of 37.1–526.6 nM, 26.4–77.4 nM, and 73.9–369.2 nM, respectively.

In our measurement of saliva secretion, a significant increase in saliva secretion was observed 60 seconds after Bet injection (Fig. 5D, blue circles). The significant increase was followed by a sustained salivary hypersecretion that lasts for 300 seconds after Bet injection.

Tear secretion level slightly increased 30 seconds after Bet injection, when compared to the level before injection (Fig. 5E, blue circles). A significant increase in tear secretion was found 60 seconds after Bet injection. The increase in tear secretion induced by Bet injection was gradually reduced until 300 seconds after injection, while maintaining a higher level of secretion when compared to that before the injection.

To confirm that YC3.60 transgenic mice reveal normal secretory function, we evaluated the pathophysiology of secretory organs in YC3.60 transgenic mice.

Gross macroscopic observation revealed no obvious difference in size and gross morphology of each secretory organ between wild-type and YC3.60 transgenic mice (Supplementary Figure 1A).

Histopathological observations showed no difference in lobe structure and acinar cell size between wild-type and YC3.60 transgenic mice (Supplementary Figure 1B).

In the wild type mice, saliva secretion and tear secretion were increased after Bet injection. No significant differences in saliva and tear secretion during 300 seconds after Bet injection were observed between YC3.60 mice and wild-type mice (Supplementary Figure 1C,D).

These results suggest YC3.60 transgenic mice reveal normal physiological function of secretory organs.

### Intracellular Ca^2+^ signaling in lacrimal gland (LG) dysfunction

Since our intravital imaging strategy was found to be successfully practicable in analyzing the intracellular Ca^2+^ signaling of secretory organs in living animals, it is reasonable to utilize this strategy to further explore the underlying cellular mechanisms of secretory dysfunctional disease in humans.

Dry eye disease, which is caused by LG dysfunction, is one of the most prevalent eye diseases^8,32,33^. The characteristic signs of this disease include tear shortage which results in ocular discomfort and visual disturbance affecting quality of life^33^.

The autonomic nervous systems consist of sympathetic and parasympathetic nerves that innervate the LG, and the parasympathetic innervation of the LG plays an essential role in the regulation of LG tear secretory function^22,34^. Therefore, we adopted post-ganglionic denervation of LG (PGDL) as a dry eye disease model to investigate the Ca^2+^ signaling in the MEC of LG under the dry eye conditions.

**Figure 6:**
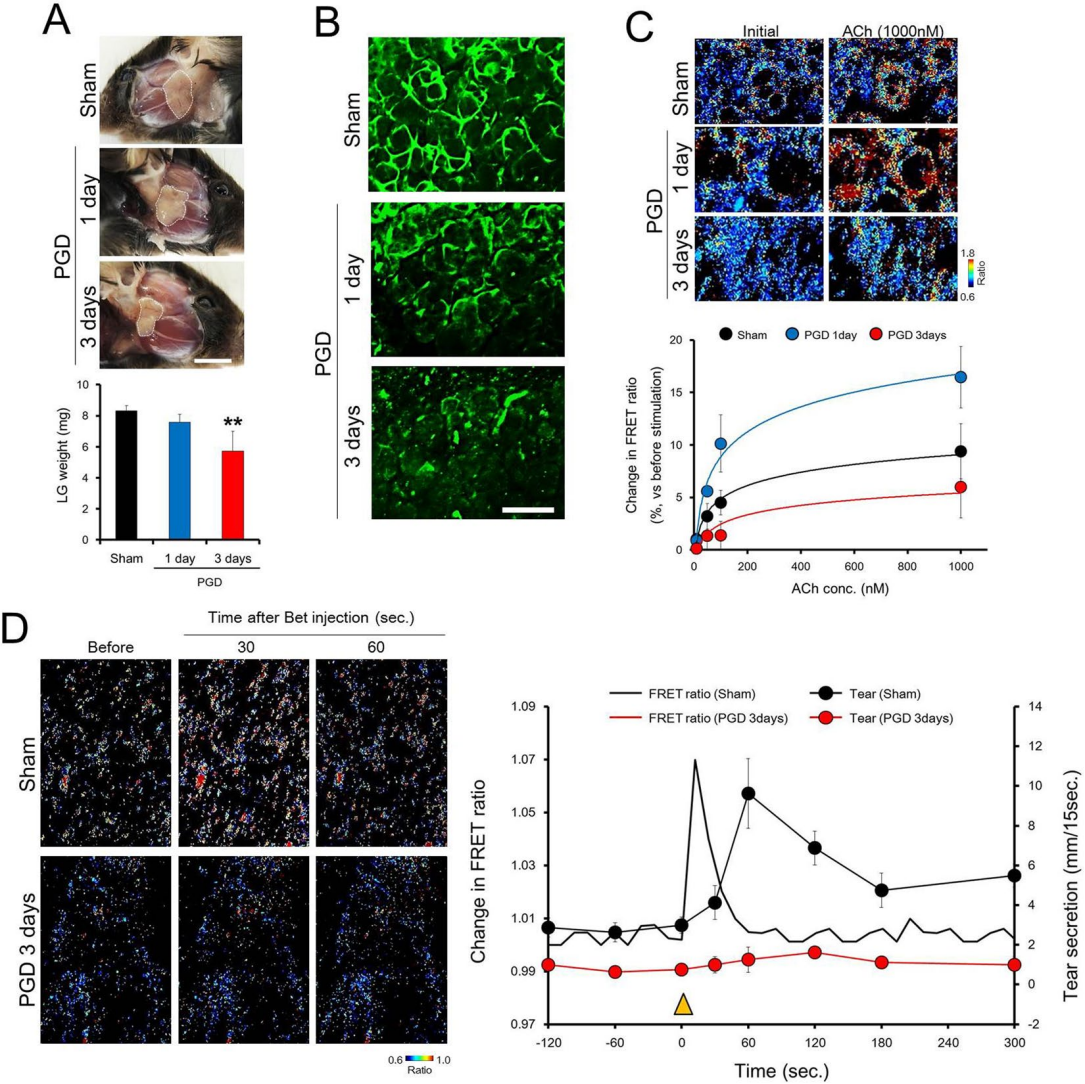
Effects of postganglionic denervation (PGD) of the lacrimal gland on Ca^2+^ response by cholinergic stimulation. (**A**) Morphological changes in the LG after PGD surgery. Upper panels show the appearance of the LG. The dotted line indicates the LG (Scale bar: 5 mm). Bar chart shows the changes in the LG. (**B**) Morphological changes in the LG myoepithelial cells after PGD surgery. The LG was immunostained with an anti-calponin antibody, which is a myoepithelial cell marker. Scale bars: 50 µm. (**C**) Changes in ACh-induced Ca^2+^ response by PGD surgery in isolated LG from YC3.60 mice. Upper panels show the pseudo-color images of the FRET ratio after stimulation with ACh (1000 nM). The line chart shows the summarized data of changes in the FRET ratio induced by ACh stimulation at doses of 1, 10, 100, and 1000 nM. Each panel shows the LG of sham surgery (upper), 1 day after PGD surgery (middle), and 3 days after PGD surgery (**A**–**C**). (**D**) Effect of PGD surgery in the intravital LG Ca^2+^ response to cholinergic stimulation. Left panels show the pseudo-color images of the FRET ratio (upper: sham, lower: 3 days after PGD surgery). The graph on the right shows the changes in the FRET ratio (line) and tear secretion (closed circles) by intravenous injection of bethanechol (Bet). Black and red are the sham and 3 days after PGD surgery, respectively. The yellow arrowhead indicates the time at which Bet was intravenously injected into mice. All data represent the mean ± SD, n = 4−5 LG or mice. **P < 0.01 versus sham.

Figure 6A shows the morphological changes of the LG after PGDL surgery. One day after the surgery, there was no obvious difference in the size of LG between the PGDL side and contralateral sham-operated side. After 3 days of PGDL, a marked reduction in the size of the LG was observed compared to the contralateral sham-operated side. The LG weight significantly decreased to approximately 75% of that of the sham-operated side after 3 days of PGDL (Fig. 6A lower).

Two-photon imaging of the LG showed an extensive decrease in the number of stellate calponin-positive MEC depending on the time after PGDL compared to the sham-operated side (Fig. 6B).

To confirm the change in Ca^2+^ responsivity to ACh stimulation in the MEC of LG, isolated LG from PGDL-treated YC3.60 transgenic mice was stimulated with ACh at a dosage of 10, 50, 100, and 1,000 nM (Fig. 6C).

In the sham-operated side, FRET ratio responses in the MEC increased upon stimulation with ACh in a dose-dependent manner. A dose-dependent increase in the FRET ratio in MEC was also observed at 1 and 3 days after PGDL. One day after PGDL, the FRET ratio elevation induced by ACh was higher than that in the sham group at any dosage. This result possibly indicated that the affinity of mAChR for ACh was increased due to acute deficiency of parasympathetic stimulation. Three days after PGDL, the ACh-induced FRET ratio elevation was lower than that of the sham group at any dosage.

Intravital Ca^2+^ imaging of LG MEC after 3 days of PGDL is shown in Fig. 6D. In the sham-operated side, the FRET ratio was elevated immediately after intravenous injection of Bet, and returned to the baseline of 60 seconds after Bet injection. Following transient elevation of the FRET ratio in LG MEC, tear secretion was markedly increased and gradually returned to baseline. In contrast, in the PGDL group, neither the FRET ratio elevation of LG MEC nor the increase in tear secretion following intravenous injection of Bet was observed. These observations suggest that a sustained deficiency of parasympathetic stimulation due to PGDL causes functional decline of LG MEC.

## Discussion

The present study verified that YC3.60 transgenic mice are useful and efficient for investigating Ca^2+^ signaling in the secretory organs *ex vivo* and intravitally.

In *ex vivo* imaging of secretory organs, we used three types of pharmacological agent to verify that YC3.60 transgenic mice are available for monitoring Ca^2+^ signaling in secretory organs. Ca^2+^ mobilization occurs in the cells by two ways, extracellular Ca^2+^ entry and intracellular Ca^2+^ release from endoplasmic reticulum. We therefore monitored the two distinct Ca^2+^ mobilization processes from initiation of Ca^2+^ increase to intracellular saturation in secretory organs during the sustained opening of membrane Ca^2+^ channel by ionomycin and endoplasmic reticulum Ca^2+^ pump by CPA, respectively. We also monitored the Ca^2+^ mobilization through physiological receptor pathway using ACh in the secretory organs and evaluated the dose-dependent Ca^2+^ elevation by ACh in pancreas and LG. Taken together with our *ex vivo* imaging verified with three pharmacological agents, we determined the availability of YC3.60 transgenic mice in analyzing the Ca^2+^ signaling in secretory organs.

Pancreatic acinar cells are a classic model of exocrine organs for investigation of secretory pathways mediated by Ca^2+^ signaling^4,24^. Although Ca^2+^ signaling in pancreatic acinar cells have been widely studied *in vitro*, only a limited number of references investigated its Ca^2+^ signaling using genetically encoded Ca^2+^ indicators (GECIs). Oshima *et al*. established a mouse line ubiquitously expressing the YC-Nano 15 probe under control of the CAG promotor and elaborated the association of Ca^2+^ signaling and exocytosis of pancreatic acinar cells^28^.

YCs are composed of a linear combination of CFP, calmodulin (CaM), a linker peptide, the CaM-binding peptide of myosin light-chain kinase (M13), and YFP. The flexibility of the linker peptide between CaM and M13 in the YCs is critical for determining Ca^2+^ sensitivity. YC-Nano15 is YC variant with a longer linker containing 5 amino acids, and has high sensitivity to Ca^2+^ ^35^. Since YC 3.60 has a short linker (2 amino acids) and a point-mutated CaM, its Ca^2+^ sensitivity is lower than YC-Nano15^35^.

Compared to YC-Nano 15, YC3.60 in pancreatic acinar cells displayed a slightly lower Ca^2+^ response to 5−10 nM ACh but higher Ca^2+^ response to 50−500 nM ACh (Supplementary Figure 2). In the LG MEC of YC 3.60 transgenic mice, the change in the FRET ratio in response to ACh was lower than that in pancreatic acinar cells at any dosage of ACh, suggesting that the sensitivity of the Ca^2+^ response to ACh in the MEC is different from that in the acinar cells. The spatial pattern of the Ca^2+^ wave induced by ACh stimulation in pancreatic acinar cells of YC3.60 transgenic mice was consistent with that of YC-Nano15 transgenic mice^28^. These results suggest that the pancreas of YC3.60 transgenic mice is a useful tool which is equally feasible as YC Nano15 transgenic mice for investigating the relationship between intravital Ca^2+^ signaling and pancreatic fluid secretion.

Nezu *et al*. determined the FRET ratio dynamics in the rat SG injected in a retrograde manner with an adenoviral vector expressing YC Nano50 via the secretory duct^29^. A great advantage in using GECI-YC3.60 transgenic mice for Ca^2+^ imaging is that it enables stable expression of the Ca^2+^ indicator in a cell type-specific manner through noninvasive delivery^13,15^. Our studies on the measurement of FRET ratio dynamics in the SG showed that Ca^2+^ signaling in distinct cell types can be simultaneously monitored *ex vivo*. Further study is needed to clarify the difference in [Ca^2+^]i responsivity to cholinergic stimulation in acinar cells and MEC.

Ca^2+^ signaling in the LG have been investigated only with isolated LG acinar cells or MEC^36,37^. No study has shown the role of the LG MEC in tear secretory function, because no tools had been developed to visualize their Ca^2+^ signaling in living animals. Interestingly, the YC3.60 transgenic mice displayed the cell-specific expression of the YC3.60 probe in the MEC of the LG. Comparative analysis of the association of intravital Ca^2+^ signaling in LG MEC and tear secretion between the healthy LG and PGD-induced dysfunctional LG indicated that Ca^2+^ signaling in MEC participates in tear-secretory function. Although the mechanism of tear-secretory function contributed by the LG is still unclear, further studies will elucidate the physiological role of MEC in tear secretion.

In conclusion, the present study showed the establishment and evaluation of an intravital Ca^2+^ visualization system using YC3.60 transgenic mice that express the Ca^2+^ indicator in specific types of cells in secretory organs to analyze the spatiotemporal dynamics of intracellular free calcium during stimulations. To further analyze the real physiological Ca^2+^ signaling in secretory organs, the application of our intravital Ca^2+^ imaging system to fiber photometry^38^ will be able to allow monitoring of Ca^2+^ signaling in secretory organs under the conditions that mice are awake and unrestraint. Thus, our study provides a novel practical basis for methodology in intravital Ca^2+^ imaging of secretory organs and will contribute to our further understanding of physiological and pathological conditions of secretory organs.

## Materials and Methods

### Animals

Eight-week-old male Yellow Cameleon 3.60 (YC3.60) transgenic mice, a mouse line expressing the Ca^2+^ sensor YC3.60 probe, and C57BL/6 J mice (wild-type) were used in this study. YC3.60 transgenic mice was obtained by crossing CAG-Cre mouse in which the Cre recombinase gene is under control of the CAG promotor (β actin chicken promotor and cytomegalovirus enhancer) and YC3.60^flox^ mouse in which neomycin phosphatase transfer gene is inserted between CAG promotor and YC3.60 gene^13,15^. All procedures were performed in accordance with the Association of Research and Vision in Ophthalmology (ARVO) statement for the Use of Animals in Ophthalmology and Vision Research and approved by the Animal Experimentation Ethics Committee of Keio University School of Medicine, Tokyo, Japan. All animals were quarantined and acclimatized for 1 week prior to the experiments under the same general conditions (room temperature was 23 ± 2 °C, humidity was 60 ± 10%, and an alternating 12-hour light-dark cycle (8 AM to 8 PM), water and food were available ad libitum).

### Visualization of cell surface and myoepithelial cells (MEC)

YC3.60 transgenic mice were euthanized with an overdose of pentobarbital sodium and the secretory organs including the pancreas, salivary gland, and lacrimal gland (LG) were dissected.

For visualization of the cell surface, these secretory organs were stained with phalloidin-labeled wheat germ agglutinin (WGA: 1:500 dilution; Vector Laboratories, Burlingame, CA, USA) which binds to lectins and acts as a marker for the cell surface^39^, at room temperature (RT) for 30 minutes. WGA-stained secretory organs were washed three times with a saline solution (140 mM NaCl, 5 mM KCl, 2 mM CaCl_2_, 1 mM MgCl_2_, 10 mM HEPES, and 10 mM dextrose [pH 7.4]) for 3 minutes.

For visualization of MEC, the secretory organs dissected from YC3.60 mice were fixed in 4% paraformaldehyde (PFA) at 4 °C overnight. After fixation, secretory organs were blocked with 1% bovine serum album (BSA) in PBS containing 0.25% Triton X-100 for 1 hour at room temperature and then incubated overnight at 4 °C with a rabbit polyclonal antibody against calponin, a specific MEC marker^16,17^ (1:300 dilution, Abcam, Cambridge, MA, USA). After being washed with PBS, secretory organs were incubated with Alexa Fluor 555-conjugated donkey-anti rabbit secondary antibody (1:300 dilution, Molecular Probes, Eugene, OR, USA) for 3 hours at room temperature (25 ± 5 °C) and washed with PBS.

### Two-photon observation of YC3.60 probe localization in secretory organs

WGA-stained or immuno-stained secretory organs were observed with an upright two-photon microscope (FV1200MPE: Olympus, Tokyo, Japan) equipped with a water immersion objective lens (XLPlaN25 × 1.05WMP, Olympus) which was connected to a femtosecond laser source, Ti:sapphire laser (MaiTaiHP: Spectra Physics, Santa Clare, Ca, USA). The excitation wavelength for YC3.60 probe, WGA, and Alexa-555 was 830 nm, and the emission was simultaneously detected through a band-pass filter for YC3.60 (510−550 nm) and for WGA and Alexa 555 (575−630 nm). Fluorescence images at a depth of approximately 200 µm from the surface of each secretory organ were reconstructed from 200 images acquired at z-step sizes of 1 µm using Imaris software (Bitplane AG, Zurich, Switzerland).

### *Ex vivo* Ca^2+^ imaging

YC3.60 transgenic mice were euthanized with an overdose of pentobarbital sodium. Each secretory organ (pancreas, SG, and LG) dissected from YC3.60 mice was transferred to round coverslips that were mounted on the bath region of a perfusion chamber and continuously perfused with a saline solution through polyethylene tubes connected to a Masterflex peristaltic pump (Cole-Parmer, Chicago, IL, USA) at a flow rate of 0.8 mL per minutes.

Solutions of ionomycin, a calcium ionophore (10 µM; Sigma-Aldrich, St. Louis, MO, USA); cycropiazonic acid (CPA, 10 µM; Sigma-Aldrich); and acetylcholine (ACh, 1 µM) were diluted to the desired concentrations with the saline solution and used as stimulants. Atropine, an antagonist for muscarinic acetylcholine receptors (1 µM, Nacalai tesque, Kyoto, Japan), was prepared in a saline solution before use. Ionomycin, CPA, and ACh were applied to each secretory organ for 4 minutes, 4 minutes and 1 minute, respectively. For evaluation of the effect of atropine on ACh-induced Ca^2+^ mobilization, atropine was applied to each secretory organ 2 minutes before the application of ACh.

Ca^2+^ signals were observed with a two-photon microscope equipped with a 25× water-immersion objective lens by measuring the change in FRET (fluorescence resonance energy transfer) ratio that was calculated as the ratio of YFP to CFP fluorescence intensity. An excitation wavelength of 830 nm was used for FRET imaging. The two-photon excited fluorescence images of CFP and YFP were acquired in separate channels through dichroic mirrors and emission filters, BP460-500 and BP520-560, respectively. In the pancreatic secretory lobe, SG and LG, the fluorescence images were acquired at approximately 1 frame/second for 8 minutes at depth-intervals of 2 µm from the surface of each secretory organ to a maximum depth of 10 µm. Fluorescence images were reconstructed using Imaris software and analyzed with MATLAB software (Math-Works, Natick, MA, USA). In the pancreatic acinar cells, the fluorescence images were acquired at approximately 1 frame/500 ms for 8 minutes. Images were analyzed with the Aqua Cosmos software (Hamamatsu Photonics, Shizuoka, Japan).

### Intravital Ca^2+^ imaging

YC3.60 transgenic mice were anesthetized with an intraperitoneal injection of urethane (1.2 g/kg). For imaging of the pancreas, mice were placed in a dorsal position. Abdominal skin and a small portion of the peritoneum located at left inferior of the sternum were incised, and then the pancreas located on the left side of the stomach was exteriorized. For imaging of the SG, the SG was surgically exteriorized after incision of the skin of the anterior neck spaces between submandibular lymph nodes and the sternum. For imaging of the LG, mice were placed in a lateral position, the skin on the right temporal side of the head was incised and the LG was exteriorized. After each secretory organ was exteriorized, a custom-built metal ring was attached to the peritoneum, sternocephalicus muscle, and masseter muscle using a cyanoacrylate-based glue (Loctite, Henkel Japan, Yokohama, Japan) for imaging of the pancreas, SG, and LG, respectively. Each exteriorized secretory organ was placed on round coverslip which is located on a custom-buit metal ring. Secretory organs were mounted with water-soluble O.C.T. compound for protecting against desiccation and then sandwiched with round coverslip for eliminating the effect of organs movement due to breathing. During intravital imaging, mouse was placed on a heating-pad and body temperature was maintained at 37 °C with thermos-controller (BWT-100, Bioresearch Center, Nagoya, Japan).

Solution of bethanechol (Bet, 1 mg/kg, Sigma-Aldrich), an agonist of the muscarinic ACh receptor, was prepared in saline solution before use. Bet was intravenously injected into jugular vein to deliver a volume up to 100 µL.

Changes in the FRET ratio were observed with a two-photon microscope equipped with an XL Fluor 4 × /340 NA 0.28 objective lens. The fluorescence images were acquired at approximately 1 frame/second for 8 minutes, at depth intervals of 5 µm from the surface of each secretory organ to a maximum depth of 50 µm. Acquired fluorescence images were reconstructed using Imaris software and analyzed with MATLAB software.

To estimate the [Ca^2+^]i in the each secretory organ, minimum FRET ratio under stimulation with Ca^2+^-free saline solution containing 2 mM EGTA (R_min_) and the maximum FRET ratio under stimulation with saline solution containing 10 µM ionomycin (R_max_) were measured. FRET ratios were converted to [Ca^2+^]i using *in situ* dissociation constant (*Kd*) (250 nM for YC3.60) and Hill coefficient (n, 1.7 for YC3.60)^14^ with standard ratiometric equation^9^; [Ca^2+^]i = *Kd*[(R − R_min_)(R_max_ − R)^−1^]^(1/n)^. Empirical value of (R_min_, R_max_) in the pancreas, SG, and LG is (1.37, 0.56), (0.75, 0.58), and (0.58, 0.50), respectively.

### Measurement of tear secretion

YC3.60 transgenic mice were anesthetized with urethane (1.2 g/kg, i.p.). Tear secretion was measured using a modified Schirmer test^37,40^ by phenol red thread (Zone-Quick; Showa Yakuhin Kako, Tokyo, Japan), which was placed on the temporal side of the conjunctiva between the limbus and the outer canthus for 15 seconds. The length of the moistened area from the edge was measured to within 0.5 mm.

The Bet solution (1 mg/kg) was used as a stimulant and intravenously injected through the jugular vein. Measurements of tear secretion were obtained 1 and 2 minutes before and 0.5, 1, 2, 3, and 5 minutes after injection of Bet.

### Measurement of saliva secretion

Saliva secretion in YC3.60 transgenic mice was measured using phenol red thread under anesthesia by urethane (1.2 g/kg, i.p.). The oral cavities were held open using a mouth gag. The retained saliva in the mouse was removed before measurement. Phenol red thread was placed at the sublingual caruncle, a mucosal fold located behind the lower incisors on the floor of the mouth^41^ under a stereomicroscope for 15 seconds. To eliminate the saliva secretion from parotid gland, cleaning tissue paper was fitted to oral cavity opposite the maxillary second molar where the excretory duct of the parotid gland opens^42^. Measurements of saliva were obtained 2 minutes before, and 1, 3, and 5 minutes after intravenous injection of Bet (1 mg/kg).

### Post-ganglionic denervation of lacrimal gland (PGDL)

PGDL was performed according to a previous report^37^. In brief, mice were placed in a prone position, and the skin on the right temporal side of the head was incised under deep anesthesia using a combination anesthetic containing medetomidine (0.75 mg/kg), butorphanol (5 mg/kg), and midazolam (4 mg/kg). The post-ganglionic nerve bundle was detached from the blood vessels at the caudal root site of the ventral surface of the LG and denervated under a stereomicroscope. PGDL was performed only on the right LG; the left LG remained as a control.

Immunohistochemistry of calponin and *ex vivo* Ca^2+^ imaging was performed 1 and 3 days after PGDL. Intravital Ca^2+^ imaging and measurement of tear secretion was performed 3 days after PGDL.

### Statistical analysis

Statistical analyses were performed with the JMP12 software (SAS Institute, Cary, NC, USA). Comparison between the two groups was determined by an F-test followed by a Student’s t-test for parametric variables and Mann-Whitey U test for non-parametric variables. Multiple comparisons were performed by a one-way ANOVA followed by parametric Dunnett test or non-parametric Steel test. Differences between the measurement variables were considered significant if the resultant P-value was 0.05 or less.

## Electronic supplementary material

Supplementary information

Supplementary Movie 1

Supplementary movie 2

Supplementary movie 3

Supplementary movie 4

Supplementary movie 5

Supplementary movie 6

Supplementary movie 7

Supplementary movie 8
