# Supplementary material for: Intravital Two-photon Imaging of Ca2+ signaling in Secretory Organs of Yellow Cameleon Transgenic Mice
Source: Sci Rep. 2018 Oct 26;8:15880. doi: 10.1038/s41598-018-34347-1 (PMC6203801; doi:10.1038/s41598-018-34347-1)
Supplement: Supplementary file 1 — Supplementary information [file 41598_2018_34347_MOESM1_ESM.pdf]

Supplementary information

Title

Intravital Two-photon Imaging of Ca^{2+} signaling in Secretory Organs of Yellow Cameleon Transgenic Mice

Full author names

Kai Jin[1,†], Toshihiro Imada[1,†], Shigeru Nakamura[1], Yusuke Izuta[1], Erina Oonishi[1], Michiko Shibuya[1], Hisayo Sakaguchi[1], Takahiro Adachi[2], Kazuo Tsubota[1]

1) Department of Ophthalmology, Keio University School of Medicine, 35 Shinanomachi, Shinjyuku-ku, Tokyo 160-8582, Japan

2) Department of Immunology, Medical Research Institute, Tokyo Medical and Dental University, 1-5-45 Yushima, Bunkyo-ku, Tokyo 113-8510, Japan

† These authors contributed equally to this work.

Supplementary Materials and Methods

Histopathological analysis

Wild-type and YC3.60 transgenic mice were euthanized with overdose of pentobarbital sodium, and their pancreas, salivary gland (SG) and lacrimal gland (LG) were dissected. The pancreas, SG and LG were fixed in a 10% formalin solution. These specimens were embedded in paraffin and cross-sectioned, Sections were subjected to hematoxylin and eosin (HE) staining. Photographs were captured using optical microscope BIOREVO BZ-9000 (Keyence, Osaka, Japan).

Measurements of saliva and tear secretion in wild type mice

Saliva secretion and tear secretion in wild type mice was measured using phenol red thread under anesthesia by urethane (1.2 g/kg). These measurements were performed as described under Methods.

Evaluation of dose dependence of the FRET ratio increase in acetylcholine (ACh) concentration in pancreatic acinar cells and LG MEC.

Acetylcholine (ACh) was applied to the pancreas and LG at doses of 5, 10, 50, 100, and 500 nM, and 1, 10, 100, and 1000 nM for 1 min, respectively. Changes in the

FRET ratio in the apical region of acinar cells were analyzed by Aqua Cosmos software (Hamamatsu Photonics, Shizuoka, Japan). The FRET ratio change in LG MEC was analyzed with Imaris software (Bitplane AG, Zurich, Switzerland) and MATLAB software (Math-Works, Natick, MA, USA).

Supplementary Figure 1

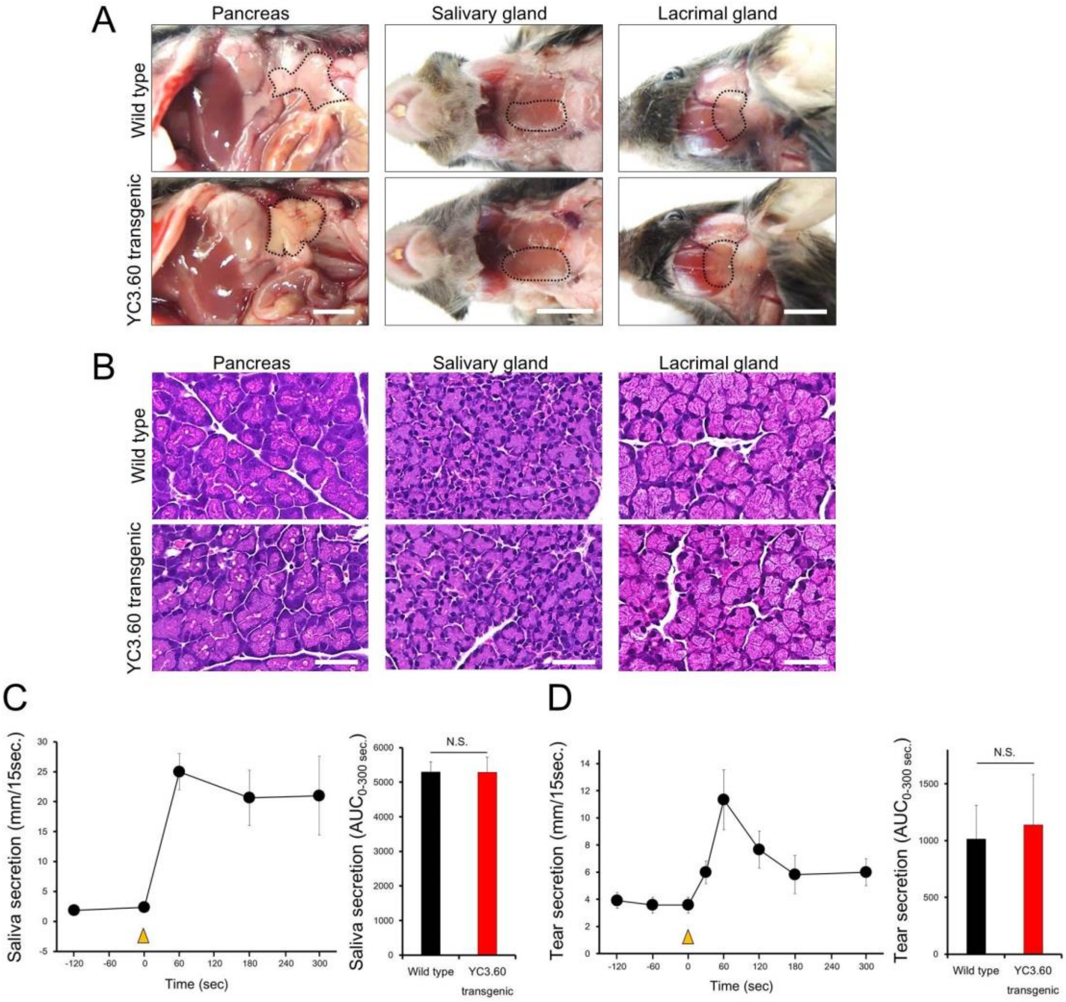

Supplementary Figure 2

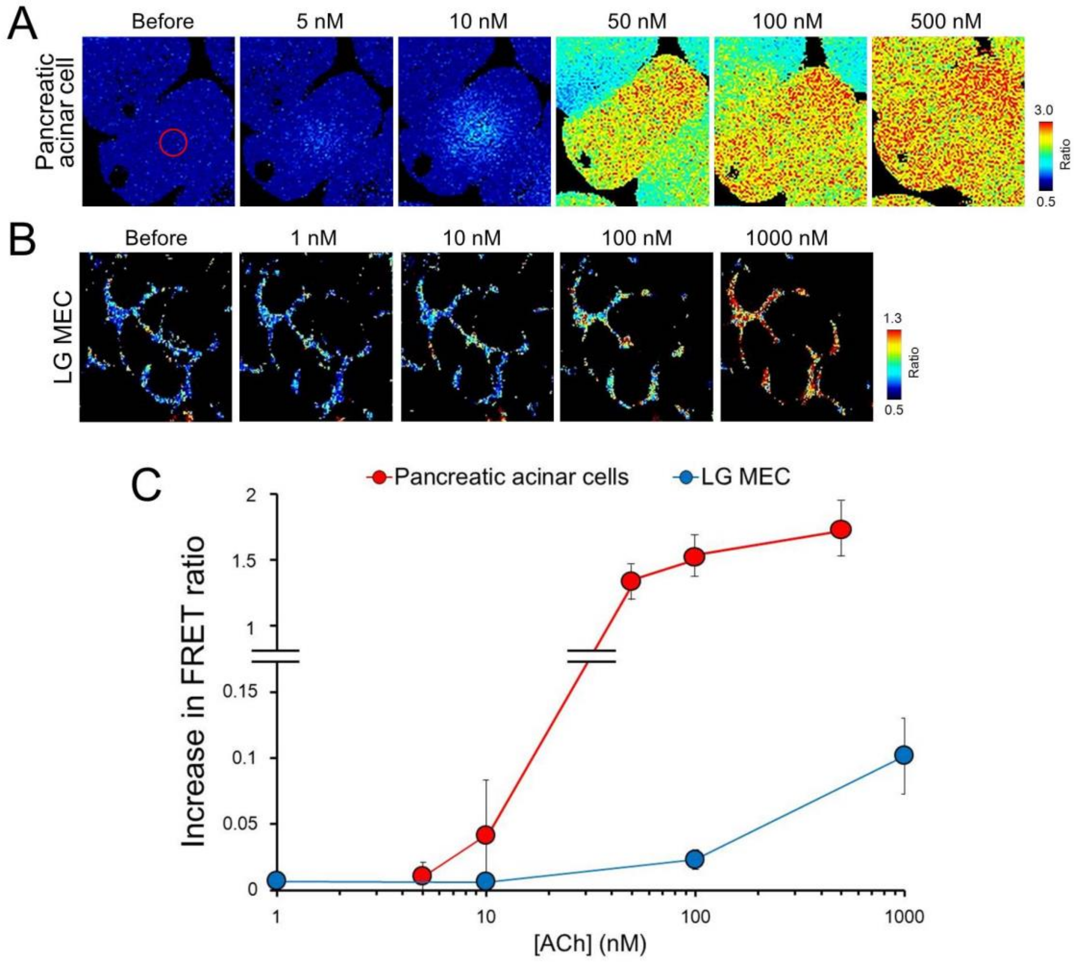

Supplementary Figure legends

Supplementary movie 1 - Three-dimensional (3D) construction of the two-photon imaging of the lacrimal gland surface from a YC3.60 transgenic mouse. The 3D movie shows the expression of the YC3.60 probe (green) and the wheat germ agglutinin (WGA, red) staining of the lacrimal gland.

Supplementary movie 2 - Changes in the FRET ratio within the pancreatic secretory lobe by ionomycin stimulation.

Supplementary movie 3 - Changes in the FRET ratio within the pancreatic acinar cell by ionomycin stimulation.

Supplementary movie 4 - Changes in the FRET ratio within the salivary gland by ionomycin stimulation.

Supplementary movie 5 - Changes in the FRET ratio within the lacrimal gland by ionomycin stimulation.

Supplementary figure 1 – Comparison of pathophysiology in secretory organs between wild-type mice and YC3.60 transgenic mice.

(A) Gross appearance of pancreas, salivary gland (SG) and lacrimal gland (LG) of wild-type mice (upper) and YC3.60 transgenic mice (lower). Scale bar is 5 mm. (B) Histopathological evaluations of pancreas, SG and LG. Upper and lower panels show wild-type and YC3.60 mice, respectively. Scale bar is 10 μm. Change in saliva secretion (C) and tear secretion (D) in wild type mice. Yellow arrow head indicate the time at which bethanechol was intravenously injected into mice. Each right bar chart shows the comparison of saliva and tear secretion between wild type (black column) and YC3.60 transgenic mice (red column), respectively. AUC of saliva and tear secretion in YC3.60 transgenic mice was calculated the data represented in Figure 5. All data represents the mean ± SD, n=4-5 mice. N.S. indicates the value not significant versus wild type mice.

Supplementary figure 2 - Dose dependence of the FRET ratio on ACh concentration in the apical region of pancreatic acinar cells.

The upper panel shows the pseudo-color image of FRET ratio induced by ACh stimulation. The red circle indicates the apical region of the acinar cells. Data represent the mean ± SD, n=12 acinar cells.

Supplementary movie 6 - Intravital Ca^{2+} imaging in the pancreas. Bethanechol (Bet) was intravenously injected into YC3.60 transgenic mice.

Supplementary movie 7 - Intravital Ca^{2+} imaging in the salivary gland. Bethanechol (Bet) was intravenously injected into YC3.60 transgenic mice.

Supplementary movie 8 - Intravital Ca^{2+} imaging in the lacrimal gland. Bethanechol (Bet) was intravenously injected into YC3.60 transgenic mice.
